# 4-Butyl­anilinium perchlorate

**DOI:** 10.1107/S1600536811022860

**Published:** 2011-06-18

**Authors:** Xing-Wei Cai, Hong-Fei Lu

**Affiliations:** a School of Biological and Chemical Engineering, Jiangsu University of Science and Technology, Zhenjiang, Jiangsu 212003, People’s Republic of China

## Abstract

In the crystal structure of the title salt, C_10_H_16_N^+^·ClO_4_^−^, the 4-butyl­anilinium cation is mirror symmetric, the butyl C atoms and anilinium N atom and 1,4-position C atoms of the benzene ring being located on the mirror plane; the perchlorate anion is also mirror symmetric, with two O atoms and one Cl atom lying on the mirror plane. Trifurcated N—H⋯O hydrogen bonding is observed between the cation and anion in the crystal structure.

## Related literature

For related amine derivatives and their applications, see: Fender *et al.* (2002 ▶); Kryatova *et al.* (2004 ▶); Fu *et al.* (2010 ▶); Aminabhavi *et al.* (1986 ▶).
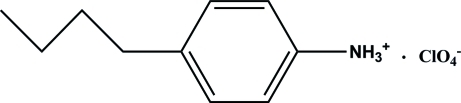


## Experimental

### 

#### Crystal data

C_10_H_16_N^+^·ClO_4_^−^
*M* *_r_* = 249.69
Monoclinic, 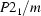

*a* = 4.8825 (10) Å
*b* = 7.9565 (16) Å
*c* = 15.452 (3) Å
β = 97.35 (3)°
*V* = 595.4 (2) Å^3^
*Z* = 2
Mo *K*α radiation
μ = 0.32 mm^−1^
*T* = 298 K
0.10 × 0.03 × 0.03 mm

#### Data collection

Rigaku Mercury2 diffractometer
Absorption correction: multi-scan (*CrystalClear*; Rigaku, 2005 ▶) *T*_min_ = 0.910, *T*_max_ = 1.000
6108 measured reflections
1466 independent reflections
1102 reflections with *I* > 2σ(*I*)
*R*_int_ = 0.064

#### Refinement

*R*[*F* ^2^ > 2σ(*F* ^2^)] = 0.084
*wR*(*F* ^2^) = 0.255
*S* = 1.15
1466 reflections
97 parameters
2 restraints
H atoms treated by a mixture of independent and constrained refinement
Δρ_max_ = 0.80 e Å^−3^
Δρ_min_ = −0.39 e Å^−3^

### 

Data collection: *CrystalClear* (Rigaku, 2005 ▶); cell refinement: *CrystalClear*; data reduction: *CrystalClear*; program(s) used to solve structure: *SHELXTL* (Sheldrick, 2008 ▶); program(s) used to refine structure: *SHELXTL*; molecular graphics: *SHELXTL*; software used to prepare material for publication: *SHELXTL*.

## Supplementary Material

Crystal structure: contains datablock(s) I, global. DOI: 10.1107/S1600536811022860/xu5241sup1.cif

Structure factors: contains datablock(s) I. DOI: 10.1107/S1600536811022860/xu5241Isup2.hkl

Supplementary material file. DOI: 10.1107/S1600536811022860/xu5241Isup3.cml

Additional supplementary materials:  crystallographic information; 3D view; checkCIF report

## Figures and Tables

**Table 1:** Hydrogen-bond geometry (Å, °)

*D*—H⋯*A*	*D*—H	H⋯*A*	*D*⋯*A*	*D*—H⋯*A*
N1—H1*A*⋯O1^i^	0.86	2.21	2.873 (8)	134
N1—H1*A*⋯O2	0.86	2.33	2.951 (7)	129
N1—H1*A*⋯O2^ii^	0.86	2.33	2.951 (7)	129
N1—H1*B*⋯O2^iii^	0.86	2.17	2.960 (4)	153

Symmetry codes: (i) 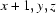; (ii) 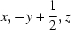; (iii) 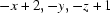.

**Fig. 1.**
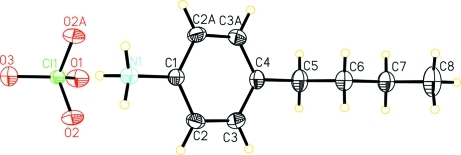
A view of the asymmetric unit with the atomic numbering scheme. The displacement ellipsoids were drawn at the 30% probability level.

**Fig. 2.**
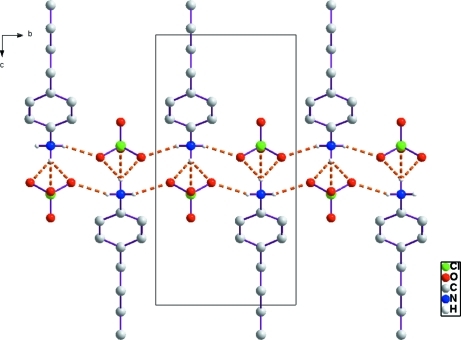
Part of the crystal packing of the title compound along the a axis. H atoms not involved in hydrogen bonding (dashed lines) have been omitted for clarity.

## supplementary crystallographic information

### Comment

The amino derivatives have found wide range of applications in material science, such as molecular recognition, fluorescence and dielectric behavior (Fender *et al.*, 2002; Kryatova *et al.*, 2004). And there has been an increased interest in the preparation of salts of amide (Aminabhavi *et al.,* 1986; Fu *et al.* 2010). We report here the crystal structure of the title compound, 4-butylanilinium monoperchlorate.

In the title compound (Fig. 1), the asymmetric unit is composed of half ClO_4_^-^ anion and half C_10_H_16_N^+^ organic cation. The N atom of the amine group is protonated. The butyl group is approximately perpendicular to the benzene plane, the torsion angle C3–C4–C5–C6 = 88.5 (6)°.

In the crystal structure, the trifurcated N—H···O hydrogen bonding is observed between the cation and anion (Table 1).

### Experimental

4-Butylanilinium perchlorate was obtained commercially from Alfa Aesar. Colourless block-shaped crystals suitable for X-ray analysis were obtained by slow evaporation of an ethanol/water (2:1 *v*/*v*) solution.

### Refinement

All H atoms attached to C atoms were fixed geometrically and treated as riding with C–H = 0.93 Å (aromatic), C–H = 0.96 Å (methyl) and C–H = 0.97 Å (methylene), with *U*_iso_(H) = 1.5*U*_eq_(C) for methyl H atoms and 1.2*U*_eq_(C) for the others. All NH_3_^+^ hydrogen atoms were calculated geometrically and were refined using a riding model with N—H = 0.86 Å and *U*_iso_(H) = 1.5*U*_eq_(N).

### Crystal data

**Table:**

C_10_H_16_N^+^·ClO_4_^−^	*F*(000) = 264
*M**_r_* = 249.69	*D*_x_ = 1.393 Mg m^−^^3^
Monoclinic, *P*2_1_/*m*	Mo *K*α radiation, λ = 0.71073 Å
Hall symbol: -P 2yb	Cell parameters from 1466 reflections
*a* = 4.8825 (10) Å	θ = 3.7–27.5°
*b* = 7.9565 (16) Å	µ = 0.32 mm^−^^1^
*c* = 15.452 (3) Å	*T* = 298 K
β = 97.35 (3)°	Block, colorless
*V* = 595.4 (2) Å^3^	0.10 × 0.03 × 0.03 mm
*Z* = 2	

### Data collection

**Table:**

Rigaku Mercury2 diffractometer	1466 independent reflections
Radiation source: fine-focus sealed tube	1102 reflections with *I* > 2σ(*I*)
graphite	*R*_int_ = 0.064
Detector resolution: 13.6612 pixels mm^-1^	θ_max_ = 27.5°, θ_min_ = 3.7°
CCD profile fitting scans	*h* = −6→6
Absorption correction: multi-scan (*CrystalClear*; Rigaku, 2005)	*k* = −10→10
*T*_min_ = 0.910, *T*_max_ = 1.000	*l* = −19→20
6108 measured reflections	

### Refinement

**Table:**

Refinement on *F*^2^	Primary atom site location: structure-invariant direct methods
Least-squares matrix: full	Secondary atom site location: difference Fourier map
*R*[*F*^2^ > 2σ(*F*^2^)] = 0.084	Hydrogen site location: inferred from neighbouring sites
*wR*(*F*^2^) = 0.255	H atoms treated by a mixture of independent and constrained refinement
*S* = 1.15	*w* = 1/[σ^2^(*F*_o_^2^) + (0.1129*P*)^2^ + 1.2735*P*] where *P* = (*F*_o_^2^ + 2*F*_c_^2^)/3
1466 reflections	(Δ/σ)_max_ < 0.001
97 parameters	Δρ_max_ = 0.80 e Å^−^^3^
2 restraints	Δρ_min_ = −0.39 e Å^−^^3^

### Special details

**Table:**

Geometry. All esds (except the esd in the dihedral angle between two l.s. planes) are estimated using the full covariance matrix. The cell esds are taken into account individually in the estimation of esds in distances, angles and torsion angles; correlations between esds in cell parameters are only used when they are defined by crystal symmetry. An approximate (isotropic) treatment of cell esds is used for estimating esds involving l.s. planes.
Refinement. Refinement of F^2^ against ALL reflections. The weighted R-factor wR and goodness of fit S are based on F^2^, conventional R-factors R are based on F, with F set to zero for negative F^2^. The threshold expression of F^2^ > 2sigma(F^2^) is used only for calculating R-factors(gt) etc. and is not relevant to the choice of reflections for refinement. R-factors based on F^2^ are statistically about twice as large as those based on F, and R- factors based on ALL data will be even larger.

### Fractional atomic coordinates and isotropic or equivalent isotropic displacement parameters (Å^2^)

**Table:**

	*x*	*y*	*z*	*U*_iso_*/*U*_eq_	
Cl1	0.6840 (3)	0.2500	0.58920 (10)	0.0330 (5)	
O3	0.7917 (12)	0.2500	0.6775 (3)	0.0577 (14)	
O2	0.7792 (8)	0.1039 (4)	0.5470 (2)	0.0512 (10)	
O1	0.3918 (11)	0.2500	0.5797 (4)	0.0584 (15)	
C1	0.8370 (13)	0.2500	0.3416 (4)	0.0338 (13)	
N1	1.0745 (12)	0.2500	0.4098 (4)	0.0408 (13)	
H1A	1.0715	0.2500	0.4653	0.061*	
H1B	1.1566	0.1543	0.4106	0.061*	
C4	0.3891 (14)	0.2500	0.2140 (4)	0.0397 (15)	
C5	0.1577 (18)	0.2500	0.1403 (5)	0.055 (2)	
H5	0.053 (13)	0.348 (8)	0.144 (4)	0.066*	
C2	0.7268 (10)	0.0998 (6)	0.3113 (3)	0.0429 (11)	
H2	0.8012	−0.0013	0.3336	0.052*	
C6	0.2653 (17)	0.2500	0.0523 (5)	0.0499 (18)	
H6	0.349 (12)	0.155 (7)	0.048 (4)	0.060*	
C7	0.043 (2)	0.2500	−0.0246 (6)	0.067 (3)	
H7	−0.091 (14)	0.344 (8)	−0.021 (4)	0.081*	
C3	0.5053 (11)	0.1013 (6)	0.2476 (3)	0.0461 (12)	
H3	0.4305	−0.0003	0.2263	0.055*	
C8	0.151 (3)	0.2500	−0.1102 (6)	0.091 (3)	
H8A	−0.0007	0.2500	−0.1562	0.136*	
H8B	0.2620	0.3485	−0.1149	0.136*	

### Atomic displacement parameters (Å^2^)

**Table:**

	*U*^11^	*U*^22^	*U*^33^	*U*^12^	*U*^13^	*U*^23^
Cl1	0.0304 (8)	0.0272 (7)	0.0404 (8)	0.000	0.0010 (5)	0.000
O3	0.071 (4)	0.054 (3)	0.045 (3)	0.000	−0.002 (2)	0.000
O2	0.057 (2)	0.0303 (18)	0.068 (2)	0.0049 (15)	0.0126 (17)	−0.0083 (16)
O1	0.035 (3)	0.049 (3)	0.090 (4)	0.000	0.006 (3)	0.000
C1	0.034 (3)	0.035 (3)	0.033 (3)	0.000	0.008 (2)	0.000
N1	0.044 (3)	0.036 (3)	0.042 (3)	0.000	0.002 (2)	0.000
C4	0.036 (3)	0.051 (4)	0.032 (3)	0.000	0.006 (3)	0.000
C5	0.048 (5)	0.069 (5)	0.046 (4)	0.000	−0.001 (3)	0.000
C2	0.048 (3)	0.031 (2)	0.049 (3)	−0.0007 (19)	0.003 (2)	0.0057 (19)
C6	0.050 (5)	0.053 (5)	0.046 (4)	0.000	0.004 (3)	0.000
C7	0.089 (7)	0.060 (5)	0.047 (5)	0.000	−0.014 (4)	0.000
C3	0.055 (3)	0.036 (3)	0.047 (3)	−0.009 (2)	0.004 (2)	−0.004 (2)
C8	0.099 (9)	0.120 (10)	0.050 (5)	0.000	−0.003 (5)	0.000

### Geometric parameters (Å, °)

**Table:**

Cl1—O3	1.398 (5)	C5—C6	1.520 (11)
Cl1—O1	1.415 (5)	C5—H5	0.94 (6)
Cl1—O2	1.439 (3)	C2—C3	1.366 (7)
Cl1—O2^i^	1.439 (3)	C2—H2	0.9300
C1—C2	1.369 (5)	C6—C7	1.503 (12)
C1—C2^i^	1.369 (5)	C6—H6	0.86 (6)
C1—N1	1.464 (8)	C7—C8	1.486 (15)
N1—H1A	0.8600	C7—H7	1.00 (7)
N1—H1B	0.8601	C3—H3	0.9300
C4—C3^i^	1.384 (6)	C8—H8A	0.9601
C4—C3	1.384 (6)	C8—H8B	0.9600
C4—C5	1.498 (10)		
			
O3—Cl1—O1	110.5 (4)	C6—C5—H5	108 (4)
O3—Cl1—O2	109.8 (2)	C3—C2—C1	118.6 (4)
O1—Cl1—O2	109.4 (2)	C3—C2—H2	120.7
O3—Cl1—O2^i^	109.8 (2)	C1—C2—H2	120.7
O1—Cl1—O2^i^	109.4 (2)	C7—C6—C5	114.2 (8)
O2—Cl1—O2^i^	107.8 (3)	C7—C6—H6	104 (4)
C2—C1—C2^i^	121.7 (6)	C5—C6—H6	107 (4)
C2—C1—N1	119.2 (3)	C8—C7—C6	113.6 (10)
C2^i^—C1—N1	119.2 (3)	C8—C7—H7	111 (4)
C1—N1—H1A	127.2	C6—C7—H7	112 (4)
C1—N1—H1B	109.6	C2—C3—C4	121.8 (5)
H1A—N1—H1B	93.1	C2—C3—H3	119.1
C3^i^—C4—C3	117.4 (6)	C4—C3—H3	119.1
C3^i^—C4—C5	121.2 (3)	C7—C8—H8A	109.3
C3—C4—C5	121.2 (3)	C7—C8—H8B	109.6
C4—C5—C6	111.5 (7)	H8A—C8—H8B	109.5
C4—C5—H5	108 (4)		
			
C3^i^—C4—C5—C6	88.1 (6)	C5—C6—C7—C8	180.0
C3—C4—C5—C6	−88.1 (6)	C1—C2—C3—C4	−0.6 (9)
C2^i^—C1—C2—C3	1.6 (10)	C3^i^—C4—C3—C2	−0.3 (10)
N1—C1—C2—C3	−180.0 (5)	C5—C4—C3—C2	176.1 (6)
C4—C5—C6—C7	180.0		

Symmetry codes: (i) *x*, −*y*+1/2, *z*.

### Hydrogen-bond geometry (Å, °)

**Table:**

*D*—H···*A*	*D*—H	H···*A*	*D*···*A*	*D*—H···*A*
N1—H1A···O1^ii^	0.86	2.21	2.873 (8)	134
N1—H1A···O2	0.86	2.33	2.951 (7)	129
N1—H1A···O2^i^	0.86	2.33	2.951 (7)	129
N1—H1B···O2^iii^	0.86	2.17	2.960 (4)	153

Symmetry codes: (ii) *x*+1, *y*, *z*; (i) *x*, −*y*+1/2, *z*; (iii) −*x*+2, −*y*, −*z*+1.
